# New Method for Differentiation of Granuloviruses (Betabaculoviruses) Based on Multitemperature Single Stranded Conformational Polymorphism

**DOI:** 10.3390/ijms19010083

**Published:** 2017-12-28

**Authors:** Martyna Krejmer-Rabalska, Lukasz Rabalski, Marlinda Lobo de Souza, Sean D. Moore, Boguslaw Szewczyk

**Affiliations:** 1 Department of Recombinant Vaccines, Intercollegiate Faculty of Biotechnology University of Gdansk and Medical University of Gdansk, 80-807 Gdansk, Poland; martyna.krejmer@biotech.ug.edu.pl (M.K.-R.); boguslaw.szewczyk@biotech.ug.edu.pl (B.S.); 2 Embrapa Recursos Genéticos e Biotecnologia, Parque Estacao Biológica, 70770-900 Brasilia, Brazil; marlinda.souza@embrapa.br; 3 Citrus Research International (CRI), P.O. Box 20285, Humewood 6013, Port Elizabeth, South Africa; seanmoore@cri.co.za; 4 Department of Zoology and Entomology, Rhodes University, P.O. Box 94, Grahamstown 6140, South Africa

**Keywords:** baculovirus detection, granulovirus detection, betabaculovirus, MSSCP, PCR, *granulin*, *late expression factor-9*

## Abstract

Baculoviruses have been used as biopesticides for decades. Recently, due to the excessive use of chemical pesticides there is a need for finding new agents that may be useful in biological protection. Sometimes few isolates or species are discovered in one host. In the past few years, many new baculovirus species have been isolated from environmental samples, thoroughly characterized and thanks to next generation sequencing methods their genomes are being deposited in the GenBank database. Next generation sequencing (NGS) methodology is the most certain way of detection, but it has many disadvantages. During our studies, we have developed a method based on Polymerase chain reaction (PCR) followed by Multitemperature Single Stranded Conformational Polymorphism (MSSCP) which allows for distinguishing new granulovirus isolates in only a few hours and at low-cost. On the basis of phylogenetic analysis of betabaculoviruses, representative species have been chosen. The alignment of highly conserved genes—*granulin* and *late expression factor-9*, was performed and the degenerate primers were designed to amplify the most variable, short DNA fragments flanked with the most conserved sequences. Afterwards, products of PCR reaction were analysed by MSSCP technique. In our opinion, the proposed method may be used for screening of new isolates derived from environmental samples.

## 1. Introduction

Due to the excessive use of chemical pesticides there is a need for finding new agents that may be useful in biological protection for both forestry and agricultural application—very specific, selective, and safe for humans. Additionally they should not accumulate in the environment and possess high virulence against insect pests [1,2]. All these features characterize members of the *Baculoviridae* family—large, enveloped, rod-shaped viruses harbouring dsDNA that infect mainly arthropod caterpillars [3,4]. The *Baculoviridae* family is divided into four genera on the basis of morphology, host taxonomy, and phylogenetic studies of genomic sequences. Baculoviruses possess 37 core genes—a set of common genes that are present in every species [5]. One of them is *late expression factor-9* (*lef-9*) encoding a RNA-polymerase subunit [6].

The Betabaculovirus genus is represented by lepidopteran-specific granuloviruses (GVs) [7,8]. They can be distinguished from nucleopolyhedroviruses (NPVs) from the other three genera (*Alphabaculovirus*, *Gammabaculovirus*, and *Deltabaculovirus*) because they are built of smaller, ovo-cylindrical occlusion bodies (OBs) made of granulin (highly conserved structural protein homolog of polyhedrin), that ranges in size from 0.12–0.35 µm in width by 0.3–0.5 µm in length [9].

Baculoviruses usually have narrow host ranges, mostly limited to one or a few closely related insect species. It is reported that around 600 species belong to the *Baculoviridae* family, while only around 90 are well described with their whole genomes deposited in GenBank database and around 20 of them are granuloviruses. Taking into account that sometimes a few baculovirus species are discovered in one insect host [10,11,12,13], there is a need for a quick, reliable, and low-cost method for screening and differentiation of new isolates/species.

Several methods for baculovirus detection in tissue cultures or environmental samples have been reported in the literature. The oldest and the simplest one employs light microscopy, which allows for visualisation of viral OBs—polyhedra for NPVs or granules for GVs. The more sophisticated method engages electron microscopy, but it is not used routinely. Other methods are associated with immunological techniques, detecting viral proteins e.g., enzyme linked immunosorbent assay (ELISA) and Western blot analysis [14,15]. Some methods allow for baculovirus DNA detection—the most popular is polymerase chain reaction (PCR—[11,13,16]) or its modifications (real-time PCR—[17]; PCR-RFLP—[18,19]). In the past few years, the emergence of next generation sequencing (NGS) methods has been observed as a way of detection and thorough characterization of new baculovirus species [20].

In the past, we described a method for detection and differentiation of NPVs [21]. In this paper, for the first time, we have proposed a method for discrimination of betabaculoviruses that can be used for screening new isolates/species of granuloviruses, which is based on the multitemperature single stranded conformational polymorphism (MSSCP) technique. Using our method we were able to differentiate all eighth tested betabaculovirus species. MSSCP is the improvement of the single stranded conformational polymorphism (SSCP) technique firstly described by Orita et al. in 1989, which used the ability of short fragments (120–250 nt) of single stranded DNA to adopt various conformations during polyacrylamide gel electrophoresis, depending on physicochemical conditions (pH, temperature, ionic strength) and the nucleotide sequence. Even one-nucleotide difference can cause the change of mobility of ssDNA [22]. The modification applied in MSSCP with respect to SSCP is based on the application of a few different temperatures changes during the gel-run, which increases the resolution of the technique.

## 2. Results

### 2.1. Phylogenetic Analysis

**Figure 1:**
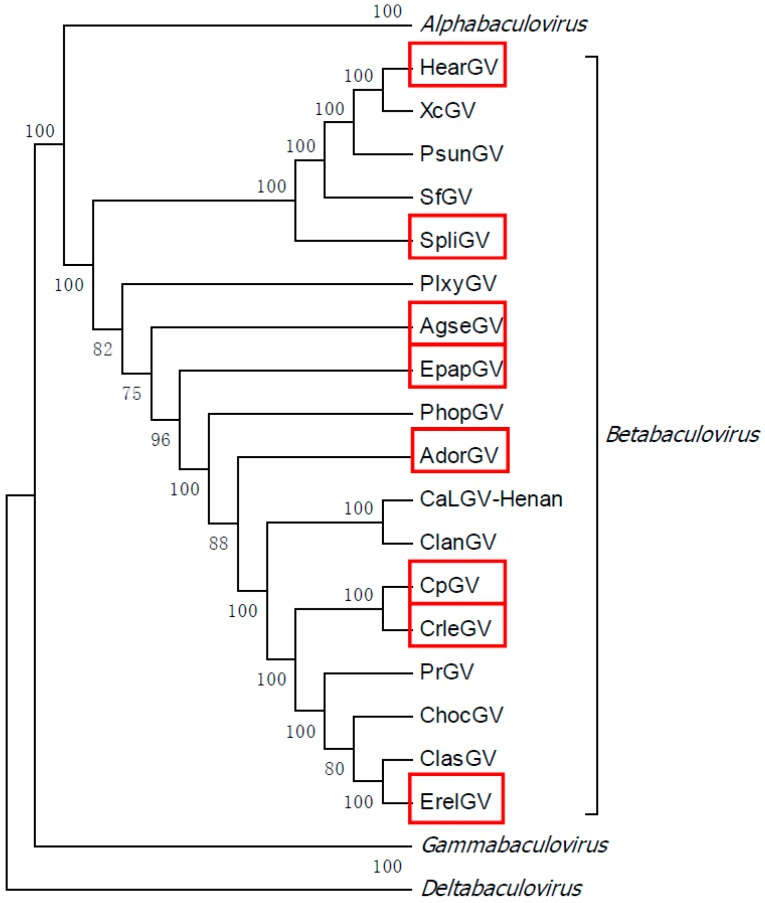
Maximum likelihood molecular phylogenetic analysis based on 37 core genes of granuloviruses. *Betabaculovirus* members (representatives of different clades) chosen for further experiments are marked in red.

The phylogenetic tree (Figure 1), that is based on amino acid sequences of proteins coding for all 37 core genes from Betabaculovirus genus and representatives of three other genera from the *Baculoviridae* family: *Alphabaculovirus*, *Deltabaculovirus,* and *Gammabaculovirus*, shows relationships among baculoviruses. The betabaculovirus sequences that are available in GenBank are listed in Table 1 with their designations and accession numbers. In the current work, we wanted to develop a method for discriminating the majority of granuloviruses species and therefore we needed to choose a representative group of these viruses They should differ significantly in genomic sequences, represent different clades among the genus, and be quite distant from each other in the phylogenetic tree, like e.g., *Helicoverpa armigera* granulovirus (HearGV) and *Epinotia aporema* GV (EpapGV). For the method to be effective in species/isolates differentiation, it should also include a pair of two closely related betabaculoviruses, e.g., *Cydia pomonella* GV (CpGV) and *Cryptophlebia leucotreta* GV (CrleGV). For the reasons stated above, we have chosen HearGV, *Spodoptera litura* GV (SpliGV), *Agrotis segetum* GV (AgseGV), EpapGV, *Adoxophyes orana* GV (AdorGV), CpGV, CrleGV, and *Erynnyis ello* GV (ErelGV) for further studies (marked in red in Figure 1).

### 2.2. Granulin and Late Expression Factor-9 Nucleotide Sequence Alignment and Primers Design for PCR Reaction Prior to Multitemperature Single Stranded Conformational Polymorphism (MSSCP)

**Figure 2:**
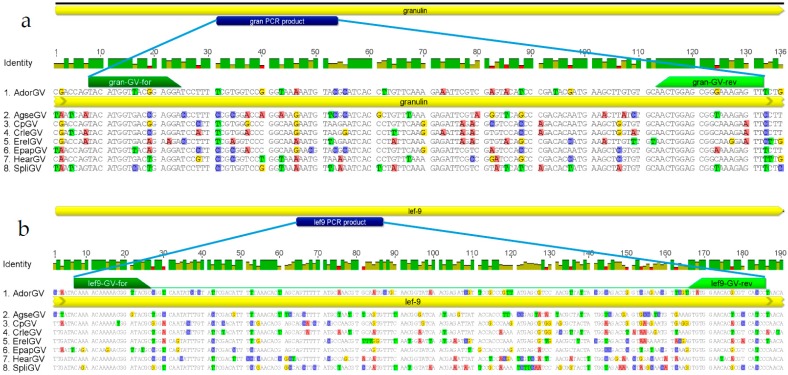
The nucleotide alignments of fragments of *gran* (**a**) and *lef-9* (**b**) from eight granuloviruses used in this study. Blue rectangles show localization of short fragments in the analyzed genes. Primer localization is marked in green. Colorful bases indicate mismatches in the alignments.

The most conserved genes are the best choice for PCR reactions prior to MSSCP technique. They are present in every genome of tested baculovirus and therefore a PCR product can be obtained in every sample that contains betabaculovirus in order to be used in the method based on MSSCP technique. MSSCP can detect minute changes in a genome, therefore a conserved genomic region that possess only a few polymorphisms that discriminate baculovirus species are preferred [23]. Usually there is more than one conformer of amplified single-stranded DNA fragment and therefore often we observe more than one electrophoretic band during MSSCP analyzes. To improve the unambiguous identification of betabaculovirus species we have chosen genes encoding granulin and late expression factor-9—both are present in all sequenced to date granulovirus genomes. These genes were extracted from whole genomic sequences available in GenBank of eight previously chosen granuloviruses (Section 2.1). The nucleotide sequences of whole gene *gran* (747 nt long) were compared. The alignment were manually analyzed in order to find a suitable place for MSSCP technique, i.e., the most conserved places for forward and reverse primer design flanking the most variable region in between 120–250 nt in size. The short fragment of 125 nt of *gran* is shown with the localization in whole gene sequence and primer binding sites (Figure 2). There is a 69–81% percentage of identity among species AdorGV, AgseGV, CrleGV, CpGV, EpapGV, ErelGV, SfGV, and SpliGV in this short fragment. The same steps were repeated for *lef-9*, which is around 1485 nt long; a short fragment chosen from this gene is 179 nt long. The lowest percentage of identity is 76% for short fragment of *lef-9* in EpapGV and ErelGV, while the highest is 93% for CpGV and CrleGV. Both sizes—125 nt and 179 nt are optimum for resolution of single stranded DNA electrophoresis (it is important to design primers in such a way that they lead to PCR products that give sharp bands in MSSCP). 

### 2.3. Multitemperature Single Stranded Conformational Polymorphism

**Figure 3:**
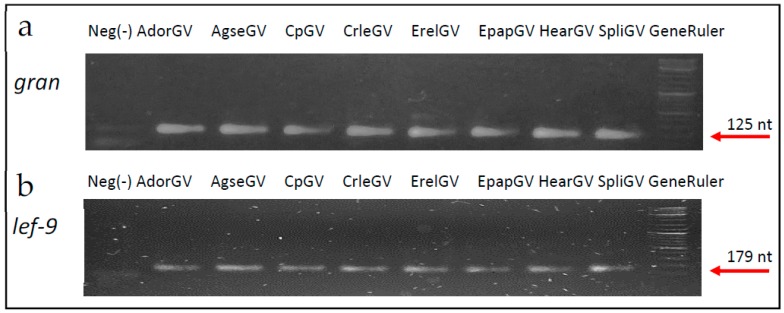
Agarose gel electrophoresis (2%) of PCR products of short fragments of *gran* (**a**)—125 nt long and *lef-9* (**b**)—179 nt long prior to MSSCP analyses. First lane—negative control (w/o DNA), lanes 2–9 granuloviruses from this study, lane 10—Gene Ruler DNA Ladder mix, size of two the lowest bands are 100 and 200 base pairs (Thermo Fisher Scientific, Waltham, MA, USA).

The two pairs of degenerate primers (Table 2) were used to amplify DNA from eight representatives of the betabaculovirus genus. Genomic DNA was isolated as described in Section 4. The PCR reactions were performed and the products possessed expected size–125 nt for *gran* and 179 nt for *lef-9* for all tested granuloviruses. Negative control (w/o DNA) was also included. The above was confirmed by agarose gel electrophoresis presented in Figure 3.

**Figure 4:**
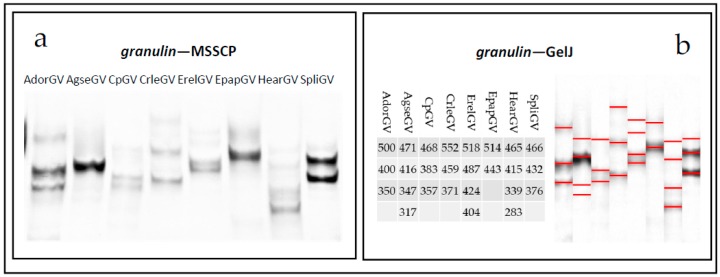
(**a**) *Granulin* gene fragments visualized with silver staining after Multitemperature Single Stranded Conformational Polymorphism analysis. (**b**) Table and picture generated by GelJ software. Different band patterns (marked with red lines) and calculated numerical fingerprints represent specific betabaculovirus species.

**Figure 5:**
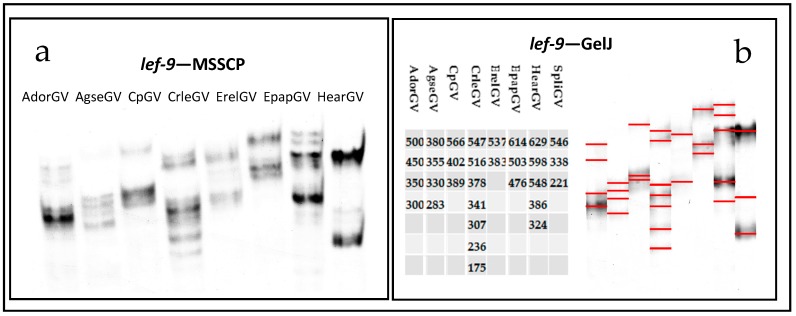
(**a**) *Late expression factor-9* gene fragments visualized with silver staining after Multitemperature Single Stranded Conformational Polymorphism analysis. (**b**) Table and picture generated by GelJ software. Different band patterns (marked with red lines) and calculated numerical fingerprints represent specific betabaculovirus species.

The amplified fragments were denatured and subjected to MSSCP polyacrylamide electrophoresis in native conditions. The gels stained with silver are presented in Figure 4a for *granulin* and Figure 5a for *lef-9*. All the tested granuloviruses have a distinct MSSCP band pattern, which correlates with their different nucleotide sequences. It is visible that band patterns in lanes differ from each other for *gran*, as well as for *lef-9*. Even for closely related species, like CpGV and CrleGV, the differences can be observed for both genes of all the tested betabaculoviruses. Each sample contained only one PCR product which was confirmed by Sanger sequencing. For fast comparison of multiple samples from numerous MSSCP runs, software like GelJ can be used [24]. Figure 4b and Figure 5b are transformed gels with the table showing a numerical pattern for every betabaculovirus species that was tested. Using an automated computational method instead of (or as complementary to) the human eye, for band pattern recognition, one can renounce any bias in human observation or interpretation. Numerical pattern was manually assigned to the first sample (based on the distance between the bands) and a computer calculated them for all the other samples. In future using, as a reference any of the previously analyzed betabaculovirus species, one can describe unknown samples.

## 3. Discussion

For decades, chemical pesticides have been used extensively, which may not only pose a threat for humans, animals, and the environment, but such an approach is not sustainable. There is therefore an urgent need to find new biological pesticides (including baculoviruses) that can successfully replace less desirable chemicals [25,26]. Consequently, fast and inexpensive methods for isolation of new biological agents are in great demand. In the past, our team has developed a method based on the MSSCP technique for detection and differentiation of nucleopolyhedroviruses [21]. In this paper, we use this technique to differentiate betabaculoviruses. Granulin and late expression factor-9 are highly conserved proteins among betabaculoviruses. Using their gene sequences in methods for differentiating species/isolates seems to be an appropriate choice. Only one day is required for obtaining results, beginning with DNA isolation, sample preparation, through PCR, and ending with a silver stained polyacrylamide gel with specific and definitive results. On the basis of the validated MSSCP method, performed in accurately defined conditions, one can determine if the identified isolate is already known or completely new, needing to be characterized with the use of more advanced technique like Next generation sequencing [27]. However, it is well known that NGS, though the most accurate technique, has some disadvantages such as time-consuming library preparation, highly qualified staff for operating the equipment and high operating costs.

One of the most valued features of MSSCP based methods is the ability to detect new, previously not characterized, species of baculoviruses. In contrast to methods based on molecular probes (like real-time PCR), analyzed sequences can be unknown. Only the flanking region needs to be recognized by degenerate PCR primers. It is possible to rapidly isolate new granulovirus species from dead caterpillars, without the need for using expensive and time consuming advanced techniques. We calculate that screening of a single sample will cost less than 2 USD (price of consumables), when 38 samples plus two positive controls are analyzed in one run. GelJ software used for data storage and gel analyzing is available free of charge.

Although baculoviruses are currently only a minor component of the plant protection market, it is estimated that 10% of the biological pesticide market is based on members of the *Baculoviridae* family [28]. One of the best known biopesticides, which is applied worldwide, comprises the codling moth granulovirus, CpGV, which protects fruit orchards, mainly apple trees, against attack by the codling moth, *Cydia pomonella* [29]. It was first identified in the codling moth in Mexico in the 1960s, characterized by high virulence and by 1995 had been registered for commercial use in crop protection. After two decades of successful application, resistance in the field occurred [30]. However, it is reported that CpGV possesses a few genetic variants that differ in virulence [31] and it is the usage of these genetic variants, other than the Mexican CpGV, in the biopesticide products that resolved the situation [31,32,33]. Similarly, there are three products containing CrleGV available on the market that vary in virulence against the false codling moth, *Thaumatotibia leucotreta*. Moreover, the virulence depends on the geographic origin of the host and its pathogen [34]. Other biological protection agents reported to be used in the field are based on PhopGV, AdorGV, PlxyGV, HomaGV (*Homona magnanina* GV), and AdhoGV [28]. As the granulovirus differentiation method based on MSSCP is inexpensive, simple, and easily implemented, it could potentially function as a screening method for finding new isolates of any of these viruses that could be used in biopesticide production. The method also allows detection of minor genetic variants that may arise during pesticide production, which could potentially compromise the efficacy of the biopesticide.

As baculoviruses can survive outside the insect host and stay viable for many years, it would be possible and beneficial to detect these viruses in the environment e.g., in soil and in foliage and so to elucidate the fate of naturally occurring baculoviruses that are beneficial in controlling important pests (e.g., in forestry—[35]). As the proposed MSSCP method is fast and sensitive, it may become a method of choice for such applications in ecosystems. A further advantage is the potential to determine whether an isolate is novel or previously described and to differentiate between the two in one run, without the need to sequence the samples.

Theoretically it is possible to find multiple virus species in a single specimen, but it is rare phenomenon. Granuloviruses have a very narrow host range—very often they infect only a single species of an insect. In such rare case, the presented method will allow for detection non-homogeneity of the sample in a single run. The software will not be able to assign any numerical pattern to the sample—this will create a “red flag” and a need for further sample analyses such as sequencing. Therefore, MSSCP is a fast preliminary method that allows for screening samples and discriminate between those that are known and unknown.

Previously, the use of capillary electrophoresis-SSCP for successful differentiation of plant virus strains was shown [36]. The described CE-SSCP method is based on relatively expensive hardware (Applied Biosystems 3130 Genetic Analyzer) and manufacturer running buffers. In contrary, the MSSCP method proposed here does not rely on advanced hardware. If the miniaturized version of the MSSCP system appears on the market (e.g., based on capillary electrophoresis) it will greatly widen the applicability of the method. To list only two advantages, the analysis time will be shorter and it will be possible to use it outside of the laboratory, directly in the field, where a sample is collected.

## 4. Materials and Methods

### 4.1. Phylogenetic Analyses of Betabaculovirus Full Genomes

The 37 core genes were extracted from each genomic nucleotide sequence of each granulovirus available in the GenBank and translated to amino acid sequences. On this basis, the phylogenetic tree of the betabaculovirus genus was constructed, utilizing the maximum-likelihood method in MEGA 7 software (accession numbers of betabaculovirus genomic nucleotide sequences are presented in Table 1). Core genes of representatives of remaining genera from the *Baculoviridae* were used in order to present the general phylogeny of the family. The percentage of 1000 bootstrap replicates in which the associated baculoviruses clustered together is shown next to the branches.

### 4.2. Virus Purification and DNA Extraction

Virus stocks were provided from the laboratories of the Authors. Crude caterpillar homogenates were filtered, then centrifuged at 5000× *g* for 10 min, the supernatant was discarded and the pellet was dissolved in a small amount of ddH_2_O. The sample was centrifuged at 5000× *g* for 10 min at room temperature (RT). The supernatant was discarded, the pellet consisting of granules was resuspended in 0.5% SDS and centrifuged at 5000× *g* for 10 min at RT. The pellet was again resuspended in 0.5 M NaCl and centrifuged as before. Thereafter, the pellet was resuspended in a small amount of ddH_2_O. All the virus stocks were dissolved in an alkaline solution (0.1 M Na_2_CO_3_, pH 10.0) for 30 min. An equal volume of Tris-HCl buffer (pH 6.4) was used for neutralization, followed by DNA extraction using MagAttract HMW DNA Kit (Qiagen, Venlo, The Netherlands), according to the manufacturer’s protocol. Isolated DNA was then checked for its quality (0.7% agarose electrophoresis) and quantity (fluorometer Quantus, Promega, Madison, WI, USA).

### 4.3. Granulin and Late Expression Factor-9 Nucleotide Sequences Alignment and Primer Design for PCR Reaction Prior to MSSCP

The genes encoding *granulin* (*gran*) and *late expression factor-9* (*lef-9*) from full nucleotide sequences of AdorGV, AgseGV, CpGV, CrleGV, EpapGV, ErelGV, HearGV, and SpliGV, available in GenBank, were extracted and two alignments were performed using Geneious Pro 7.1 default settings (Figure 2). The most conserved places in the nucleotide sequences of those genes were chosen in order to design degenerate pairs of primers. In the MSSCP method, it is important to find suitable places where the most conserved sequences flank the most variable ones around 120–250 nt in size. One set of common primers for all tested species was designed for each gene. Less degenerate primers produced sharper bands in polyacrylamide gel electrophoresis used for MSSCP.

### 4.4. Polymerase Chain Reaction (PCR)

DNA templates from eight granuloviruses were subjected to PCR with the use of GoTaq G2 Hot Start Green Master Mix (Promega, Madison, WI, USA) according to the manufacturer protocol (1× GoTaq G2 Hot Start Master Mix (2×); 0.5 μM forward, 0.5 μM reverse primer, DNA template 1 μL, nuclease-free water up to 25 μL). PCR primer sequences are presented in Table 2. PCR conditions were as follows: *lef-9*—95 °C 3 min; (95 °C 30 s, 46 °C 30 s, 72 °C 15 s) × 27; 72 °C 30 s; *gran*—95 °C 3 min; (95 °C 30 s, 37.7 °C 30 s, 72 °C 10 s) × 27; 72 °C 30 s. PCR products were loaded onto agarose electrophoresis (2%).

### 4.5. Multitemperature Single Stranded Conformational Polymorphism (MSSCP) Method

For MSSCP analysis, 3 μL of DNA was added to 10 μL of denaturing buffer (0.1 M NaOH and 10 mM EDTA) and incubated at 98 °C for 5 min. The samples were immediately cooled on ice and, prior to loading on the gel, 3 μL of dye solution (0.1% bromophenol blue, 0.1% xylene cyanol in formamide) was added. The mixture was immediately loaded onto 14% native polyacrylamide gel (37.5:1 acrylamide to bis-acrylamide ratio). Electrophoresis was carried out in 0.5× TBE (45 mM Tris, 45 mM boric acid, 1 mM EDTA, and pH 8.0) in the DNA Pointer System (BioVectis, Warsaw, Poland) at four different temperatures. The MSSCP electrophoresis conditions for *lef-9* were: 5 °C 600 Vh, 12 °C 600 Vh,19 °C 600 Vh, 29 °C 500 Vh and for *gran* were: 5 °C, 15 °C, 25 °C, 35 °C for 420 Vh each. Before transferring samples onto the gel, a 100 V × h 9 °C preelectrophoresis was performed. After electrophoresis, gels were stained using the Silver Stain Kit from BioVectis (Poland) and analyzed using GelJ software with default settings [24].

## 5. Conclusions

Alignment of nucleotide sequences of *granulin* (*gran*) and *late expression factor-9* (*lef-9*) derived from 17 members of the *Betabaculovirus* genus were performed. Representatives of different clades from the phylogenetic tree were chosen for design of a universal set of primers for PCR reaction in order to amplify the short fragment of *gran* and *lef-9*. Two pairs of degenerate primers were chosen to amplify short fragments of *granulin* and *lef-9* genes from betabaculoviruses in PCR reactions in order to detect granulovirus DNA in environmental samples prior to MSSCP. A fast and inexpensive method based on the MSSCP technique has been developed for granulovirus differentiation. On the basis of different band (and/or numerical) patterns, it was possible to conclude whether the baculovirus isolated from a caterpillar is a novel isolate/species or one that is already known. Using automated software like GelJ it is possible to create a database where information about all baculovirus isolates could be stored and compared between laboratories.

## Figures and Tables

**Table 1:** Betabaculoviruses included in the phylogenetic analyses (Figure 1) for which genomes are available in GenBank database with their accession numbers and abbreviations.

Baculovirus Designation	Betabaculovirus	Accession Number	Baculovirus Designation	Betabaculovirus	Accession Number
AdorGV	*Adoxophyes orana* GV	NC_005038	ErelGV	*Erynnyis ello* GV	KJ406702
AgseGV	*Agrotis segetum* GV	NC_005839	HearGV	*Helicoverpa armigera* GV	NC_010240
ChocGV	*Choristoneura occidentalis* GV	NC_008168	PhopGV	*Phthorimaea operculella* GV	NC_004062
ClanGV	*Clostera anachoreta* GV	NC_015398	PrGV	*Pieris rapae* GV	NC_013797
ClasGV	*Clostera anastomosis* GV	KR091910	PlxyGV	*Plutella xylosetella* GV	NC_002593
CaLGV-Henan	*Clostera anastomosis* GV (Henan isolate)	NC_022646	PsunGV	*Pseudaletia unipuncta* GV	NC_013772
CrleGV	*Cryptophlebia leucotreta* GV	NC_005068	SfGV	*Spodoptera frugiperda* GV	NC_026511
CpGV	*Cydia pomonella* GV	NC_002816	SpliGV	*Spodoptera litura* GV	NC_009503
EpapGV	*Epinotia aporema* GV	NC_005839	XcGV	*Xestia c-nigrum* GV	NC_002331

**Table 2:** Primers used in PCR reactions to amplify short fragments of *gran* and core gene—*lef-9.* B—C or G or T; N—any nucleotide; R—A or G; Y—C or T; H—A or C or T.

Gene	Forward	Reverse
*gran*	5′TACATGGTBACNGARGA3′	5′AAYTCYTTNCCGCTCCAGTT3′
*lef-9*	5′CARAACAARAAYGGRTAYGC3′	5′GGRTGNCGHGTGTTCCAYAC3′

## Abbreviations

*gran*	*granulin*
*lef-9*	*late expression factor-9*
MSSCP	Multitemperature Single Stranded Conformation Polymorphism
NGS	Next Generation Sequencing
nt	nucleotide
OBs	occlusion bodies
PCR	Polymerase Chain Reaction
RFLP	Restriction Fragment Length Polymorphism
ssDNA	single stranded DNA
